# Generation of functional hepatocytes from human spermatogonial stem cells

**DOI:** 10.18632/oncotarget.7092

**Published:** 2016-01-31

**Authors:** Zheng Chen, Min Sun, Qingqing Yuan, Minghui Niu, Chencheng Yao, Jingmei Hou, Hong Wang, Liping Wen, Yun Liu, Zheng Li, Zuping He

**Affiliations:** ^1^ State Key Laboratory of Oncogenes and Related Genes, Renji- Med X Clinical Stem Cell Research Center, Ren Ji Hospital, School of Medicine, Shanghai Jiao Tong University, Shanghai 200127, China; ^2^ Department of Urology, Ren Ji Hospital, School of Medicine, Shanghai Jiao Tong University, Shanghai Institute of Andrology, Shanghai 200001, China; ^3^ Shanghai Key Laboratory of Assisted Reproduction and Reproductive Genetics, Shanghai 200127, China; ^4^ Shanghai Key Laboratory of Reproductive Medicine, Shanghai 200025, China

**Keywords:** human spermatogonial stem cells, transdifferentiation, hepatic stem cells, mature and functional hepatocytes

## Abstract

To generate functional human hepatocytes from stem cells and/or extra-hepatic tissues could provide an important source of cells for treating liver diseases. Spermatogonial stem cells (SSCs) have an unlimited plasticity since they can dedifferentiate and transdifferentiate to other cell lineages. However, generation of mature and functional hepatocytes from human SSCs has not yet been achieved. Here we have for the first time reported direct transdifferentiation of human SSCs to mature and functional hepatocytes by three-step induction using the defined condition medium. Human SSCs were first transdifferentiated to hepatic stem cells, as evidenced by their morphology and biopotential nature of co-expressing hepatocyte and cholangiocyte markers but not hallmarks for embryonic stem cells. Hepatic stem cells were further induced to differentiate into mature hepatocytes identified by their morphological traits and strong expression of CK8, CK18, ALB, AAT, TF, TAT, and cytochrome enzymes rather than CK7 or CK19. Significantly, mature hepatocytes derived from human SSCs assumed functional attributes of human hepatocytes, because they could produce albumin, remove ammonia, and uptake and release indocyanine green. Moreover, expression of β-CATENIN, HNF4A, FOXA1 and GATA4 was upregulated during the transdifferentiation of human SSCs to mature hepatocytes. Collectively, human SSCs could directly transdifferentiate to mature and functional hepatocytes. This study could offer an invaluable source of human hepatocytes for curing liver disorders and drug toxicology screening and provide novel insights into mechanisms underlying human liver regeneration.

## INTRODUCTION

Allogeneic liver transplantation has been regarded as an effective treatment for the end-stage and inherited liver diseases [1, 2]. However, severe shortage of liver donors restricts the clinical applications of liver transplantation [3]. As examples, there are 18,000 patients waiting for liver transplantation in the United States of America, but only 5,000 donated livers are available per year [1]. As a result, numerous patients with liver disorders die before liver transplantation can be performed. Human hepatocyte transplantation has been suggested as a promisingly alternative therapy for liver-related diseases [4]. It has been reported that a number of patients could recover the function of liver through human fetal hepatocyte transplantation [5, 6]. In addition, hepatocyte transplantation may possess several advantages over the whole-liver transplantation: i) it is technically simple; ii) it can facilitate multiple treatment of a single patient; and iii) it is “reversible” since the native liver does not need to be removed [6]. Unfortunately, the availability of primary human hepatocytes is rather limited, and these cells are hard to proliferate and have short-term viability *in vitro* [7]. Therefore, it is urgently required to seek an ideal cell source from stem cells and/or extra-liver tissues to generate mature and functional human hepatocytes for treating patients with the end-stage and/or inherited liver diseases. In addition to the therapeutic application, generation of human hepatocytes from stem cells and human other tissues could be utilized for liver disease modeling as well as drug and toxicity screening.

Stem cells have recently become the most promising source of hepatocytes. A number of studies have shown that hepatocytes can be derived from embryonic stem (ES) cells, mesenchymal stem cells, and the induced pluripotent stem (iPS) cells [8–10]. The transplantation of hepatocytes derived from stem cells can recover liver damage [11–13]. However, there are certain hurdles and unresolved risk before the eventual usage of these stem cells in clinic, e.g., ethical issues with ES cells, tumorigenesis and the risk of virus infection associated with the iPS cells [2]. Thus, it is essential to search for a readily available source from adult stem cells for cell-based therapy of human hepatocytes. Spermatogonial stem cells (SSCs) have an unlimited plasticity since they can dedifferentiate and transdifferentiate to other cell lineages. However, the generation of mature and functional hepatocytes from human SSCs *in vitro* has not yet been achieved.

SSCs are a subpopulation of type A spermatogonia in mammalian testis [14]. Increasing evidence has demonstrated that SSCs from both mouse and human testes can acquire pluripotency and can dedifferentiate into ES-like cells which subsequently differentiate into various cell lineages of three germ layers [15–18]. Nevertheless, the ES-like cell stage is adverse to clinical application due to potential tumorigenesis. Notably, it has been shown that mouse SSCs could transdifferentiate into prostatic, uterine, and skin epithelium without the ES-like cell stage *in vivo* [19]. In this study, we proposed a novel concept that human SSCs can directly transdifferentiate to mature and functional hepatocytes, which achieved two significant endpoints. First of all, direct transdifferentiation of SSCs to human hepatocytes without the process of dedifferentiation to ES-like cells and embryonic body formation could simplify the reprogramming procedure. Secondly and importantly, our direct transdifferentiation using growth factors and hormone without gene transduction could be much safer to generate mature and functional human hepatocytes for cell therapy of liver diseases. Here we present a detailed induction protocol as well as molecular and cellular evidence supporting direct transdifferentiation of human SSCs to the cells with morphological, phenotypic and functional features of mature human hepatocytes. Significantly, our ability of generating mature and functional human hepatocytes from patients’ SSCs could provide an invaluable and new cell source for the treatment of liver diseases without ethical issues and immune rejection. This study also sheds a new insight into molecular mechanisms underlying liver development and regeneration.

## RESULTS

### Identification and characterization of human SSCs

**Figure 1:**
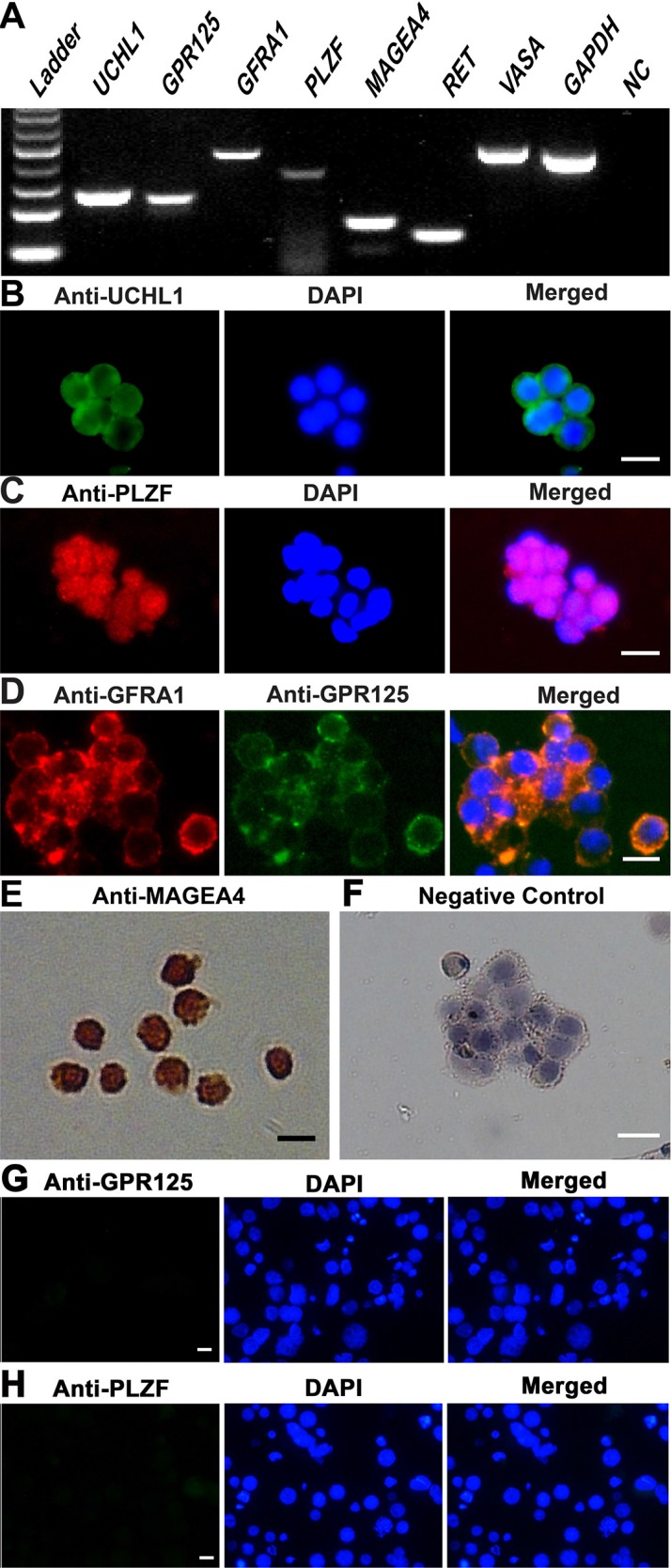
Characterization and identification of human SSCs (**A**) RT-PCR revealed the expression of *UCHL1*, GPR125, *GFRA1*, *PLZF*, *MAGEA4*, *RET*, and *VASA* in the freshly isolated human male germ cells. *GAPDH* was used as a loading control of total RNA, whereas RNA without RT but with PCR served as a negative control (NC). (**B**–**H**) Immunocytochemistry showed the expression of phenotypic markers, including UCHL1 (**B**), PLZF (**C**), GPR125 and GFRA1 (**D**), and MAGEA4 (**E**) in the freshly isolated GPR125-positive cells as well as GPR125 (**G**) and PLZF (**H**) in GPR125-negative cells. Replacement of primary antibodies with isotype IgGs was used as a negative control (**F**). Scale bars in B–H = 10 μm.

Human SSCs were separated by a two-step enzymatic digestion and magnetic-activated cell sorting (MACS) using an antibody against GPR125 pursuant to the method as previously described [20]. The identity of freshly isolated cells was characterized using various markers for male germ cells and SSCs. RT-PCR showed that the transcripts of *VASA*, *GPR125, UCHL1, PLZF, GFRA1, RET*, and *MAGEA4* were present in the freshly isolated cells (Figure 1A). RNA without RT but PCR with *GAPDH* was used a negative control (NC), and no PCR product was seen in these cells (Figure 1A), thus confirming the specific expression of these genes in the freshly isolated human male germ cells. Immunocytochemistry revealed that UCHL1 (Figure 1B), PLZF (Figure 1C), and MAGEA4 (Figure 1E) were expressed in the freshly isolated human male germ cells. Double immunostaining further displayed that GFRA1 and GPR125 were co-expressed in these cells (Figure 1D). Replacement of primary antibodies with isotype IgGs, and no immunostaining was observed in the freshly isolated cells (Figure 1F), thus verifying specific staining of these proteins in these cells. In addition, the expression of GPR125 (Figure 1G) and PLZF (Figure 1H) was undetected in GPR125-negative cells by MACS. Together, these results suggest that the freshly isolated human male germ cells are phenotypically human SSCs.

### Direct transdifferentiation of human SSCs to hepatic stem cells

**Figure 2:**
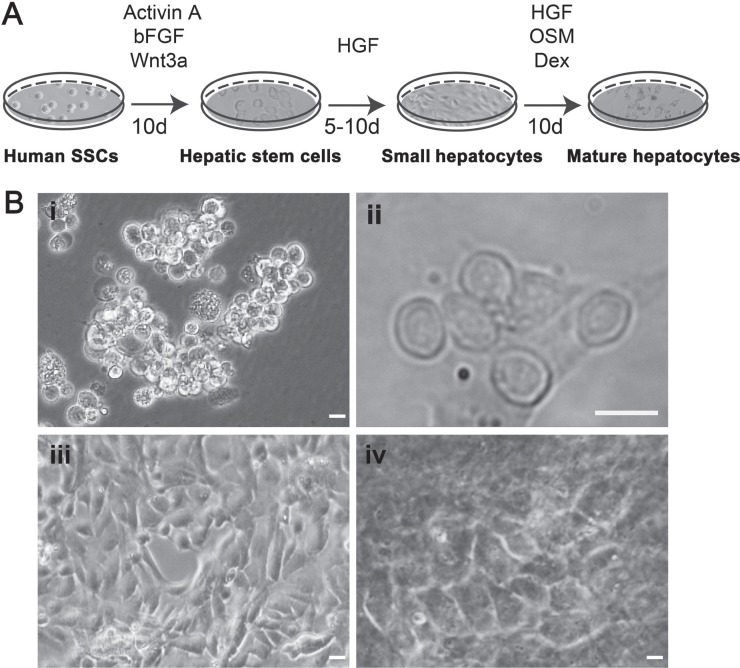
The procedure for transdifferentiation of human SSCs to mature hepatocytes and their morphological features (**A**) Schematic diagram showed the procedures for inducing human SSCs to transdifferentiate to mature hepatocytes. (**B**) Morphology was characterized for human SSCs (i), hepatic stem cells (ii), small hepatocytes (iii), and mature hepatocytes (iv). Scale bars in i–iv = 10 μm.

To induce the transdifferentiation of human SSCs to hepatic stem cells, we mimicked the condition for liver embryonic development using the conditioned medium containing Activin A, bFGF, and Wnt3a (Figure 2A). After 10 days of induction, human SSCs proliferated slightly (Figure 2B-i), and their morphology were changed and became oval and stereoscopic in shape (Figure 2B-ii). Under phase-contrast microscope, the cells assumed a higher ratio of nuclei to cytoplasm and few organelles, which was similar to hepatic oval cells (termed hepatic stem cells).

**Figure 3:**
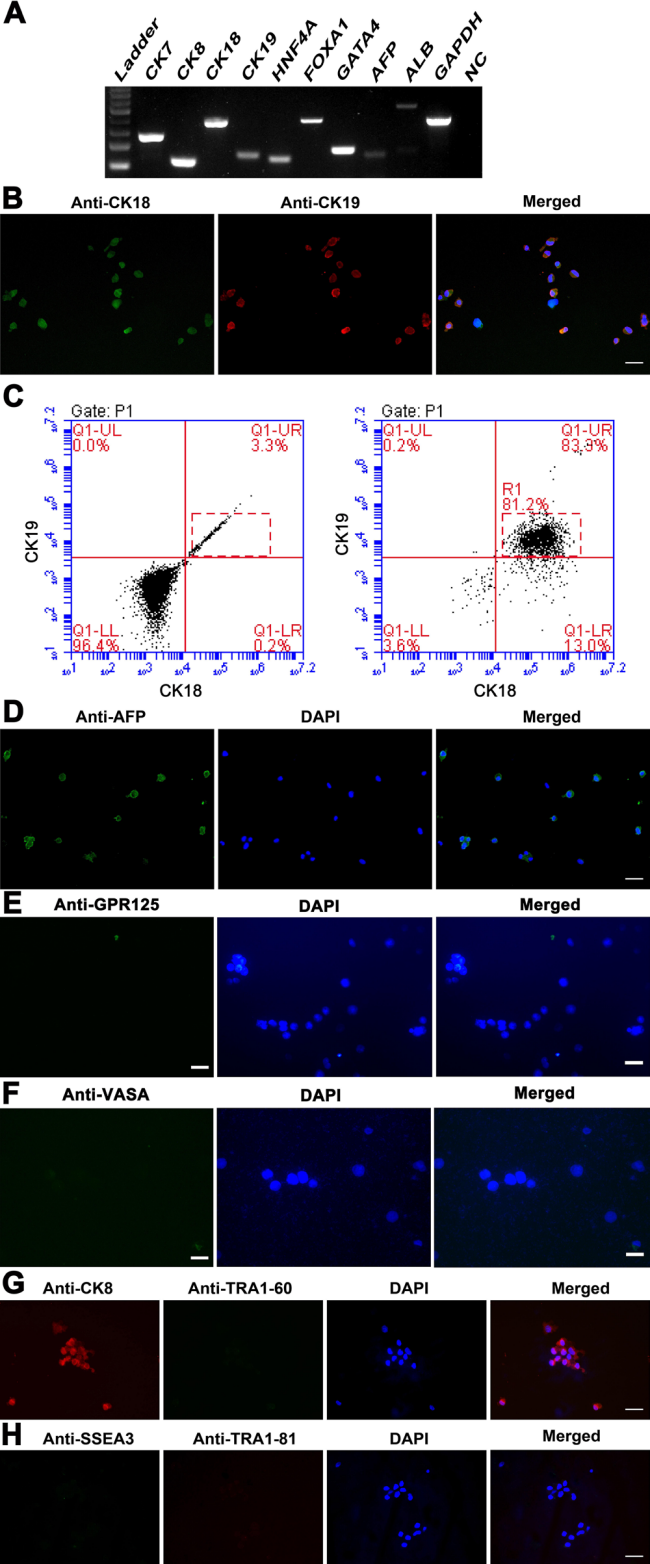
Phenotypic characteristics of transdifferentiation of human SSCs into hepatic stem cells at transcription and translation levels (**A**) RT-PCR displayed the expression of specific genes for hepatic stem cells, including *CK7*, *CK19*, *CK8*, *CK18*, *HNF4A*, *FOXA1*, *GATA4*, *AFP*, and *ALB* in the cells derived from human SSCs. *GAPDH* served as loading controls of total RNA. (**B**) Immunocytochemistry showed CK18 and CK19 expression in the cells derived from human SSCs. Scale bar in B = 20 μm. (**C**) Flow cytometry revealed the percentage of CK18- and CK19- positive cells in the transdifferentiated cells from human SSCs (right panel). Replacement of primary antibodies with PBS served as a negative control (left panel). (**D**–**H**) Immunocytochemistry showed protein expression of AFP (**D**), GPR125 (**E**), VASA (**F**), CK8 and TRA1-60 (**G**), and SSEA3 and TRA1-81 (**H**) in the cells obtained from human SSCs. Scale bars in D–H = 20 μm.

**Figure 4:**
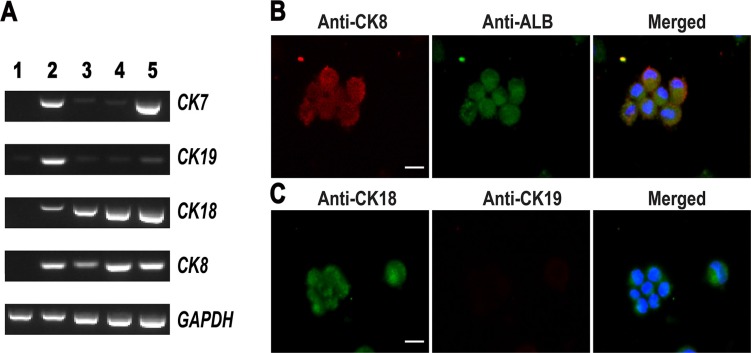
Phenotypic characterization of small hepatocytes derived from human SSCs (**A**) RT-PCR displayed the transcripts of *CK8*, *CK18*, *CK7*, and *CK19* in human SSCs (lane 1), human hepatic stem cells (lane 2), small hepatocytes (lane 3), mature hepatocytes (lane 4), and human hepatocyte line L02 cells (lane 5). (**B**–**C**) Immunocytochemistry showed the co-expression of CK8 and ALB (B) as well as the expression of CK18 and CK19 (C) in the cells derived from human SSCs. Scale bars in B–C = 10 μm.

We next examined the phenotypic characteristics of the cells induced from human SSCs at transcriptional and translational levels in order to clarify their identities. RT-PCR analyses showed that the cells derived from human SSCs expressed both hepatocytic and cholangiocytic specific genes, e.g., *CK7*, *CK19*, *CK8*, *CK18*, *ALB*, *HNF4A*, *FOXA1*, *GATA4*, and *AFP* (Figure 3A, Figure 4A, lane 2). Double immunostaining displayed that over 80% of the cells derived from human SSCs were co-expressing CK18 (a marker for hepatic cells) and CK19 (a hallmark for biliary epithelial cells) (Figure 3B), thus demonstrating their bipotency in nature. The high conversion rate was consistent with our data of flow cytometry showing that 81.2% of the cells derived from human SSCs were positive for both CK18 and CK19 (Figure 3C, right panel). Immunocytochemistry further revealed that AFP, a specific marker of hepatic stem cells, was highly expressed in the cells derived from human SSCs (Figure 3D). In addition, GPR125 (Figure 3E) and VASA (Figure 3F) were hardly or undetected in these cells derived from human SSCs. Collectively, these data implicate that human SSCs were converted into hepatic stem cells.

We asked whether human SSCs dedifferentiated into ES-like cells during the conversion process. It has been reported that mouse and human SSCs can dedifferentiate into ES-like cells which subsequently produce endoderm lineages of cells [15–18]. The ES-like cells derived from SSCs acquire ES cell properties, since they are positive for OCT4, SSEA4, SSEA3, TRA1–60, and TRA1–81 [15–18]. Notably, we didn't detect the expression of ES cell markers, including TRA1–60 (Figure 3G), SSEA3 and TRA1–81 (Figure 3H), in the cells derived from human SSCs, reflecting that human SSCs didn't dedifferentiate into ES-like cells and that they directly transdifferentiated to hepatic stem cells.

### Differentiation of human SSCs-derived hepatic stem cells into hepatocytes

**Figure 5:**
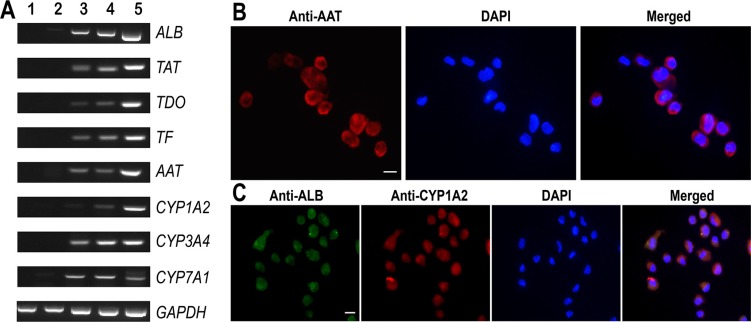
Phenotypic characteristics of mature hepatocytes derived from human SSCs (**A**) RT-PCR displayed the transcripts of *ALB*, *TAT*, *TDO*, *TF*, *AAT*, *CYP1A2*, *CYP3A4*, *CYP7A1* in human SSCs (lane 1), human hepatic stem cells (lane 2), small hepatocytes (lane 3), mature hepatocytes (lane 4), and human hepatocyte line L02 cells (lane 5). *GAPDH* served as loading controls of total RNA. (**B**–**C**) Immunocytochemistry showed protein expression of AAT (B) as well as co-expression of ALB and CYP1A2 (C) in the cells derived from human SSCs. Scale bars in B–C = 10 μm.

Hepatic stem cells are bipotent since they can differentiate to both hepatic lineage and biliary epithelial cells. We made endeavors to further induce the differentiation of hepatic stem cells derived from SSCs using hepatocyte culture medium (HCM) supplemented with HGF. After 5–10 days of induction (Figure 2A), hepatic stem cells derived from human SSCs could proliferate and they changed the morphology similar to small hepatocytes (Figure 2B-iii). In addition, we assessed biochemical phenotype of these cells to probe their identity. As shown in Figure 4A, lane 3, the cells derived from hepatic stem cells expressed both *CK8* and *CK18* transcripts at high levels, whereas *CK19* and *CK7* mRNA was significantly reduced in these cells, which implicates that under HCM and HGF induction hepatic stem cells differentiated to small hepatocytes but not biliary lineage cells. Immunocytochemistry further confirmed RT-PCR results. As shown in Figure 4B, the cells derived from hepatic stem cells co-expressed ALB and CK8 proteins. Double immunostaining revealed that CK18 protein was detected in the cells derived from human SSCs (Figure 4C), and in contrast, CK19 protein was undetected in these cells (Figure 4C). Furthermore, the cells derived from human hepatic stem cells expressed a number of hepatocyte-specific genes at low levels, e.g., *ALB*, *TDO*, *TF*, *AAT*, *TAT*, and cytochrome P450 (CYP450) enzymes including *CYP1A2, CYP3A4* and *CYP7A1* (Figure 5A, lane 3). Taken together, these data demonstrate that the hepatic stem cells derived from human SSCs differentiated to small hepatocytes but not cholangiocytes.

### Generation of human mature hepatocytes from human SSCs

It has been reported that HGF, oncostain M (OSM) and dexamethasone (Dex) promote hepatocyte maturation [21]. Therefore, we employed the combination of HGF, OSM and Dex to further facilitate the maturation of small hepatocytes. After 10 days of treatment (Figure 2A), the cells changed their morphology and became polygonal appearance (Figure 2B-iv), which was similar to the morphology of primary human hepatocytes. We next probed the expression of specific hepatic markers in the cells at both transcriptional and translational levels. As shown in Figure 4A, lane 4, high levels of CK8 and CK18 but not CK7 or CK19 were observed in the cells derived from human SSCs. Notably, the cells derived from primary human SSCs had strong mRNA expression of *ALB*, *TAT*, *TDO*, *TF*, *AAT*, *CYP1A2*, *CYP3A4*, and *CYP7A1* (Figure 5A, lane 4). Furthermore, immunocytochemistry by single and double staining revealed that the cells derived from human SSCs induced by HGF, OSM and Dex were positive for AAT (Figure 5B), ALB and CYP1A2 (Figure 5C). Considered together, these results suggest that small hepatocytes derived from human SSCs differentiated into mature hepatocytes with morphological and phenotypic features.

### Mature hepatocytes derived from human SSCs assumed functional attributes of human hepatocytes

**Figure 6:**
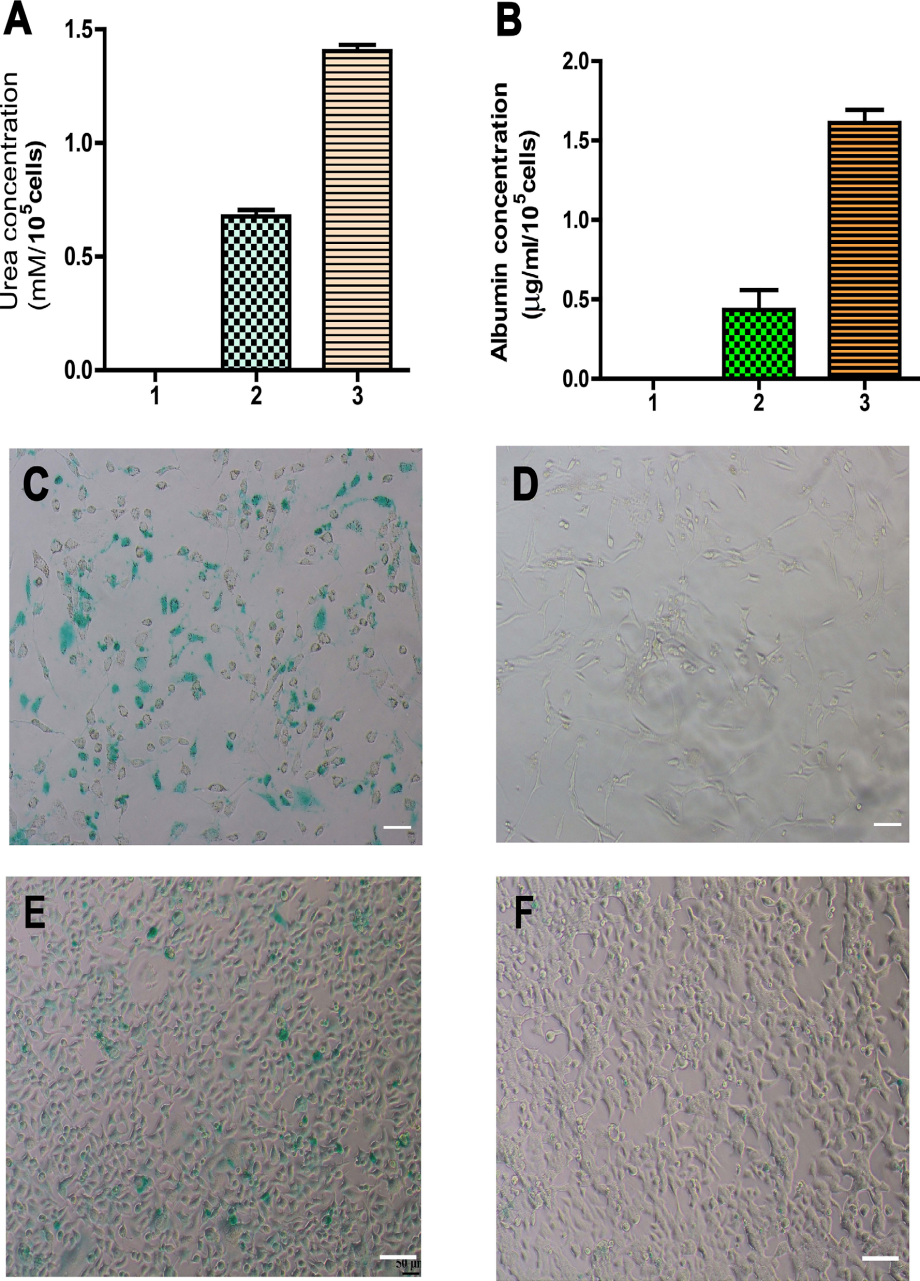
Function assays of albumin synthesis, urea productions, and cellular uptake and release of ICG of mature hepatocytes derived from human SSCs (**A**) Urea assay showed urea production in human SSCs (lane 1), mature hepatocytes derived from human SSCs (lane 2), and human hepatocyte line L02 cells (lane 3). (**B**) ELISA showed albumin synthesis in human SSCs (lane 1), mature hepatocytes derived from human SSCs (lane 2), and human hepatocyte line L02 cells (lane 3). (C–F) Cellular uptake (**C**) and release (**D**) of ICG in mature hepatocytes derived from human SSCs. The uptake (**E**) and release (**F**) of ICG in human hepatocyte line L02 cells were used as positive controls. Scale bars in C–F = 50 μm.

We measured albumin synthesis, urea production, and indocyanine green (ICG) uptake and release to test whether mature human hepatocytes derived from human SSCs assumed functional attributes of hepatocytes. Urea production is a characteristic of mature hepatocyte function. Urea assay showed that the differentiated cells from human SSCs could remove ammonia from culture medium (Figure 6A). Albumin synthesis assay is a specific test for the presence and metabolic activity of mature hepatocytes. As shown in Figure 6B, the cells derived from human SSCs possessed the capacity to produce albumin (0.43 ± 0.07 μg/ml/10^5^ cells), although albumin production was relatively lower than human hepatocyte line L02 cells. The ICG test has been used to examine the function of mature hepatocytes to assess their ability of excretion for endogenous and exogenous compounds. As shown in Figure 6C, ICG-positive staining was clearly seen in the cells derived from human SSCs. Notably, the ICG uptake in human SSCs-derived hepatocytes was excreted by 12 hours after removal of ICG from culture medium (Figure 6D). The uptake and release of ICG from mature hepatocytes was comparable to those of human hepatocyte line L02 cells (Figure 6E–6F). In parallel, ICG uptake was not observed in human germline stem cell line [22] without HGF, OSM or Dex treatment (data not shown). Collectively, our results clearly indicate that the cells derived from human SSCs possess functional attributes of primary human hepatocytes.

### The expression ofβ-CATENIN, transcript factors and cell cycle regulators during the transdifferentiation of human SSCs into functional hepatocytes

**Figure 7:**
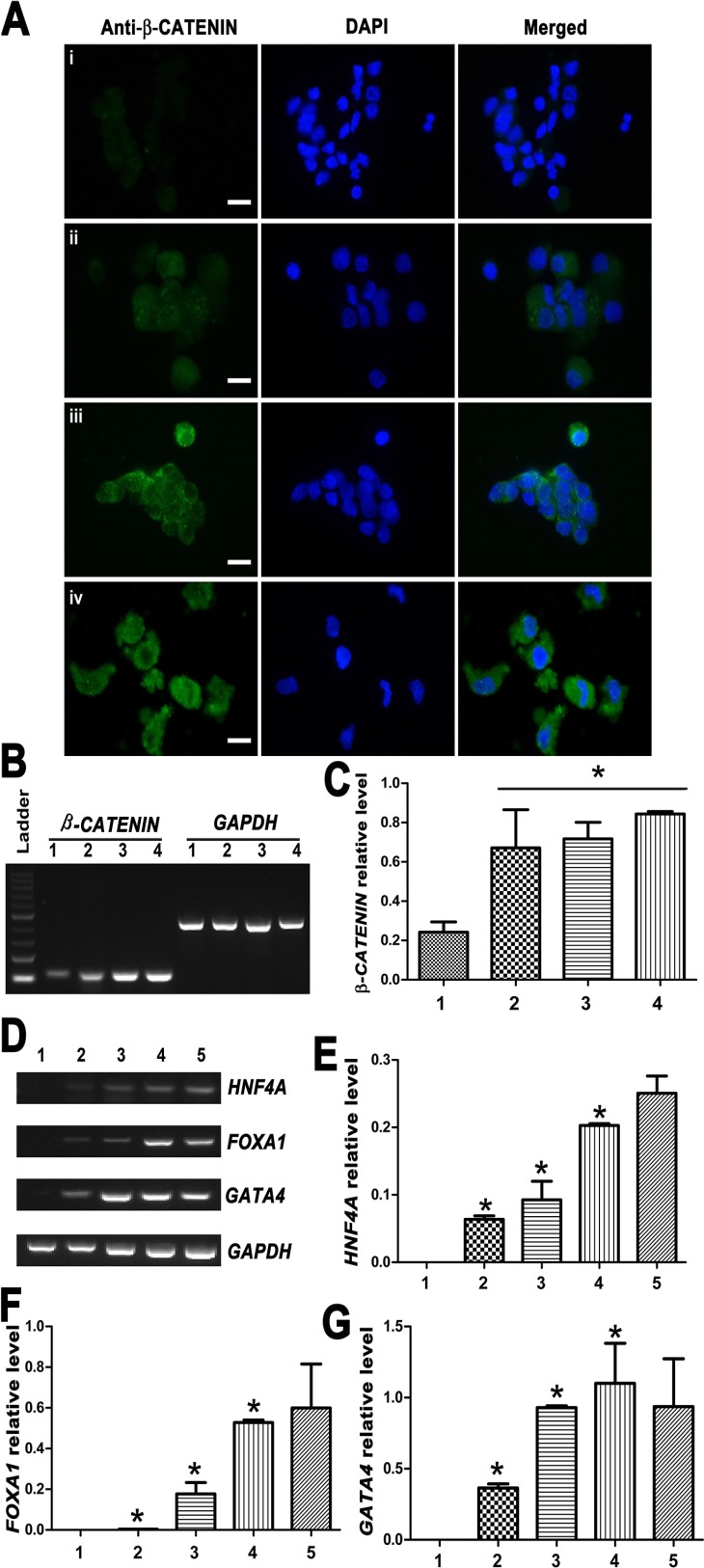
Expression changes of β-CATENIN and transcription factors HNF4A, FOXA1 and GATA4 during the transdifferentiation of human SSCs to mature hepatocytes (**A**) Immunocytochemistry revealed the expression of β-CATENIN in human SSCs (i), hepatic stem cells (ii), small hepatocytes (iii), and mature hepatocytes (iv). Scale bars = 10 μm (**B**–**G**) RT-PCR showed the transcripts of *β-CATENIN* and transcription factors *HNF4A*, *FOXA1* and *GATA4* during the conversion of human SSCs to mature hepatocytes. Lane 1: human SSCs, lane 2: hepatic stem cells, lane 3: small hepatocytes, lane 4: mature hepatocytes, lane 5: human hepatocyte line L02 cells. *indicated statistically significant differences (*p* < 0.05) among human SSCs, hepatic stem cells, small hepatocytes, and mature hepatocytes.

To gain novel insights into molecular mechanisms underlying the transdifferentiation of human SSCs into mature hepatocytes, we asked what signaling pathway was involved during this conversion process. As shown in Figure 7A, immunocytochemistry showed that the expression of β-CATENIN protein was upregulated in hepatic stem cells, small hepatocytes, and mature hepatocytes derived from human SSCs compared to human SSCs. RT-PCR further revealed that *β-CATENIN* transcript was enhanced remarkably during the conversion process (Figure 7B–7C). Furthermore, the expression of transcription factors *HNF4A*, *FOXA1* and *GATA4*, was increased gradually from human SSCs to mature hepatocytes (Figure 7D–7G).

**Figure 8:**
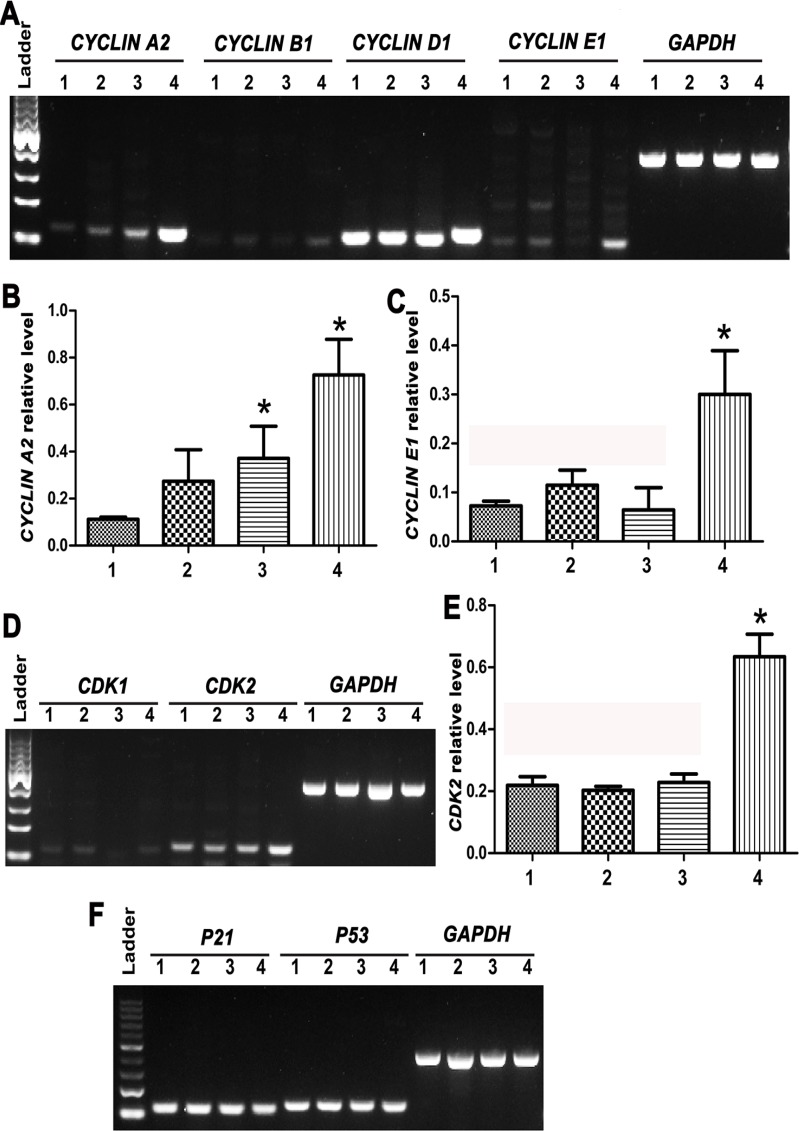
Expression changes of cell cycle regulators *CYCLIN A2, CYCLIN B1, CYCLIN D1, CYCLIN E1, CDK1/2, P21 and P53* during the transdifferentiation of human SSCs to mature hepatocytes (**A**–**F**) RT-PCR showed the transcripts of *CYCLIN A2*, *CYCLIN B1*, *CYCLIN D1*, *CYCLIN E1*, *CDK1, CDK2*, *P21*, and *P53* in human SSCs (lane 1), hepatic stem cells (lane 2), small hepatocytes (lane 3), and mature hepatocytes (lane 4). *indicated statistically significant differences (*p* < 0.05) between mature hepatocytes or small hepatocytes and human SSCs.

We further examined expression changes of *CYCLIN A2*, *CYCL* IN B1, *CYCLIN D1*, *CYCLIN E1*, *CDK1*, *CDK2*, *P53* and *P21* transcripts during the transdifferentiation of human SSCs to mature hepatocytes. As shown in Figure 8A–8F, RT-PCR analysis revealed that *CYCL* IN A2, *CYCLIN E1* and *CDK2* transcripts were enhanced significantly in mature hepatocytes compared to human SSCs, hepatic stem cells and small hepatocytes, suggesting that these cell cycle proteins are involved in hepatocyte maturation. No obvious change in the transcripts of *CYCLIN B1, CYCLIN D1, CDK1*, *P21* and *P53* was observed during the conversion of human SSCs to mature hepatocytes.

## DISCUSSION

It has been reported that hepatic stem cells can differentiate into hepatocytes [23–25]. However, it is hard to obtain human hepatic stem cells due to the scarcity of human liver tissues. Therefore, more attention has been paid to generate hepatocytes from extra-liver source and other adult stem cells. We have previously shown that mouse SSCs could directly transdifferentiate into mature hepatocytes using the conditioned medium [26]. Notably, there are distinct differences in cell types and phenotype of SSCs between human and mice [20]. With regard to the identity of SSCs, A_dark_ and A_pale_ spermatogonia have been regarded as human SSCs, whereas A_s_ spermatogonia are generally considered the actual mouse SSCs. There are also phenotypic differences in SSCs between human and rodents. As an example, POU5F1 (Oct-4) is a marker of mouse SSCs, whereas it is not present in human SSCs [20]. Similarly, KIT is regarded as a marker for mouse differentiating spermatogonia, but it is undetected in human spermatogonia [20]. To generate functional and mature cells from human SSCs has certain advantages over other cells. Firstly, human SSCs assume unlimited potentials compared to other adult stem cells, since they can acquire pluripotency to become ES-like cells. Secondly, human SSCs can transdifferentiate to cell lineages of other tissues, highlighting their great plasticity. Thirdly, human SSCs have no ethical concern associated with human ES cells. Finally, there is no risk of virus infection and tumorigenesis to utilize human SSCs compared with human iPS cells. Here we have for the first time demonstrated that human SSCs could directly transdifferentiate to the cells with morphological, phenotypic and functional attributes of human mature hepatocytes.

We first isolated and purified human SSCs from the testis tissues of OA patients using enzymatic digestion and MACS and verified the identity of these cells using a variety of markers for germ cells and SSCs. VASA has been recognized as a maker for germ cells [27], while MAGEA4 and UCHL1 are hallmarks for spermatogonia [28]. GFRA1 and RET are co-receptors for GDNF and they have been generally regarded as markers for SSCs [29, 30], while GPR125 and PLZF are considered hallmarks for human SSCs [20]. We found that the freshly isolated human male germ cells expressed VASA, UCHL1, MAGEA4, GFRA1, RET, GPR125 and PLZF, implicating that these cells are actually human SSCs in phenotype.

We mimicked the condition for liver development to induce the transdifferentiation of human SSCs to functional hepatocytes. It has been reported that canonical Wnt pathway is involved in specifying definitive endoderm and it induces direct conversion of non-hepatic endodermal cells into hepatocytes [31], and bFGF is essential to initiate the onset of hepatic development [32]. Activin A can efficiently induce endoderm differentiation of human ES cells [33]. Wnt3a, bFGF and Activin A were thus selected to coax the transdifferentiation of human SSCs into the cells with morphological and phenotypic characteristics of hepatic stem cells. In morphology, the cells derived from human SSCs resembled hepatic stem cells, since they were oval and stereoscopic in appearance and they possessed stem cell properties. In phenotype, the cells derived from human SSCs expressed specific genes of hepatic stem cells, including *CK7*, *CK19*, *CK8*, *CK18*, *ALB*, and *AFP*. Immunocytochemistry further confirmed the co-expression of CK18 and CK19 of these cells, which are hallmarks for hepatic cells and bile epithelial cells, respectively. Taken together, these results at transcription and translation levels suggest that the cells derived from human SSCs assume biopotential nature of hepatic stem cells. Significantly, the conversion of human SSCs to hepatic stem cells was relatively efficient using our induction procedure compared to human ES cells or iPS cells, because more than 80% of human SSCs could be converted into human hepatic stem cells, as evidenced by our double immunostaining and flow cytometry analyses. Generation of mature hepatocytes from human ES cells seems to be inefficient due to the low purity (70%) and large cellular heterogeneity [34], while hepatocytes derived from iPS cells have a limited regeneration of the liver [35]. It is worth noting that the cells derived from human SSCs didn't express the ES cell markers, including SSEA3, TRA1-60, and TRA1-81, suggesting that human SSCs directly transdifferentiated to hepatic stem cells without dedifferentiation to ES-like cells, which is essential for simplifying differentiation procedure and improving safety for their clinical applications.

Hepatic stem cells are biopotential since they can differentiate to both biliary epithelial cells and hepatocytes [36]. HGF is required for liver development and it is associated with the ontogenesis of liver [37]. Hepatocyte differentiation can be induced from mesenchymal stem cells by HGF and other growth factors [38]. Here we found that HGF and HCM could induce hepatic stem cells derived from human SSCs to differentiate into small hepatocytes. In phenotype, the cells derived from human SSCs expressed the hallmarks for hepatocytes, including ALB, TAT, TDO, AAT, TF, and CYP450 enzymes CYP1A2, CYP3A4 and CYP7A1, whereas the expression of CK19 and CK7 (markers for bile epithelial cells) was remarkably reduced or undetected. Small hepatocytes were further induced to become mature in HCM supplemented with OSM and Dex. In morphology, the cells derived from human SSCs became polygonal with a low nucleus/cytoplasm ratio. Under phenotype, these cells expressed numerous hepatocyte markers, including *ALB*, *TAT*, *TDO*, *TF*, *AAT*, *CYP1A2*, *CYP3A4*, and *CYP7A1*, and they were negative for CK19 or *CK7*, hallmarks for bile epithelial cells. Notably, the cells derived from human SSCs possessed numerous functional properties of primary human hepatocytes, e.g., albumin synthesis, ammonia clearance, and uptake and release of ICG. Taken together, these data indicate that human SSCs can directly transdifferentiate to cells with morphology, phenotype and function of human mature hepatocytes.

It remains unknown about what signaling pathways are activated in the transdifferentiation process of human SSCs to mature hepatocytes. Wnt/β-catenin signaling pathway has been suggested to play a crucial role in liver development [39]. It has been reported that Wnt/β-catenin signaling is required for liver specification, hepatoblast proliferation and differentiation, and conditional knockout of β-catenin in hepatoblast results in underdeveloped liver [40, 41]. Here we found that the expression of β-CATENIN and its downstream targets including *HNF4A, FOXA1* and *GATA4* was enhanced during the transdifferentiation of human SSCs into functional hepatocytes, suggesting that β-CATENIN/HNF4A/*FOXA1/GATA4* signaling pathway might be involved in this conversion process. In addition, we found that *CYCLIN A2*, *CYCLIN E1* and *CDK2* expression was upregulated in mature hepatocytes compared to human SSCs, hepatic stem cells and small hepatocytes, suggesting that these cell cycle proteins may be involved in the maturation of hepatocytes. Together, these findings shed a novel insight into molecular mechanisms underlying cell reprogramming and human liver development. Recently, it has been shown that wild-type p53-induced phosphatase 1 (Wip1) plays an important role in liver regeneration [42, 43]. Nevertheless, our data indicated that the expression of *P53* and *P21* were not changed during the conversion of human SSCs to mature hepatocytes, suggesting that P53 and P21 are not involved in this transdifferentiation process.

## CONCLUSION

In summary, we have for the first time demonstrated that human SSCs can directly transdifferentiate into hepatic stem cells that are capable of differentiating into the cells with morphology, phenotype and functional attributes of mature hepatocytes. Significantly, our ability of generating mature and functional human hepatocytes from patients’ own SSCs could avoid immunological rejection. This study thus provides a novel strategy to generate mature and functional human hepatocytes for cell therapies for liver diseases and drug toxicology screening and offers new signaling pathways underlying the development of human liver and cell reprogramming.

## MATERIALS AND METHODS

### Procurement of testicular biopsies from obstructive azoospermic (OA) patients

Testicular biopsies were obtained from OA patients with microdissection and testicular sperm extraction at Ren Ji Hospital affiliated to Shanghai Jiao Tong University School of Medicine. All OA patients had normal spermatogenesis, and they were caused by inflammation and vasoligation rather than by congenital absence of the vas deferens or other diseases including cancer.

This study was approved by the Institutional Ethical Review Committee of Ren Ji Hospital (license number of ethic approval: 2012–01), Shanghai Jiao Tong University School of Medicine, and an informed consent of testicular tissues for research only was obtained from all subjects. All experiments were performed in accordance with relevant guidelines and regulation of the Institutional Ethical Review Committee of Ren Ji Hospital.

### Isolation of human spermatogonial stem cells (SSCs)

Testicular tissues from OA patients were washed exclusively in Dulbecco modified Eagle medium (DMEM) (Gibco) with antibiotics containing penicillin and streptomycin (Gibco) to remove potential contamination of Leydig cells and myoid cells. Human SSCs were isolated using a two-step enzymatic digestion and MACS according to procedure as previously described [20]. Briefly, human seminiferous tubules were isolated from testicular tissues by the first enzymatic digestion utilizing 2 mg/ml collagenase IV (Gibco) and 1 μg/μl DNase I (Gibco) in 34°C water bath for 10–15 min. Male germ cells were further isolated from seminiferous tubules using a second enzymatic digestion with 4 mg/ml collagenase IV, 2.5 mg/ml hyaluronidase (Sigma), 2 mg/ml trypsin (Sigma), and 1 μg/μl DNase I for 15 min and followed by differential plating [20]. For differential plating, cell mixture suspension was seeded into in 10-cm diameter tissue culture dishes in DMEM/F-12 supplemented with 10% fetal bovine serum (FBS) (Gibco) and 10^3^ units/ml leukemia inhibitory factor (LIF, Sigma) and incubated at 34°C in 5% CO_2_ for 12 hours. When Sertoli cells attached to the dishes, human male germ cells remained in suspension and they were collected by centrifuging at 1000 rpm for 5 min. Human male germ cells were incubated with GPR125 (Abcam) at 4°C overnight. After washes with BSA-EDTA-PBS (phosphate-buffered saline) buffer, the cells were suspended in 80 μl upon buffer supplemented with 20 μl goat anti-rabbit IgG magnetic microbeads (Miltenyi Biotec) and they were incubated at 4°C for less than 20 min. After incubation, 400 μl of BSA-EDTA-PBS buffer were added to the cells to get a final volume of 500 microliters. The cell suspension was loaded on the column, and GPR125-negative cells were first collected. The column was removed from the magnet, and GPR125-positive human SSCs were collected through washing with BSA-EDTA-PBS buffer.

### Transdifferentiation of human SSCs to hepatic stem cells and mature hepatocytes

The protocol for transdifferentiating human SSCs to mature hepatocytes included three steps. First of all, human SSCs were cultured at a density of 1,000 cells/well in 24-well plates with DMEM/F12 containing 10% KSR, 100 ng/ml Activin A (Peprotech, Rocky Hills, NJ), 50 ng/ml Wnt3a (R & D System), and 20 ng/ml bFGF (Peprotech). The conditioned medium was changed every 2 days. After 10 days of induction, this media was replaced with HCM plus EGF supplement (BD Bioscience, Bedford, MA) and 20 ng/ml hepatocyte growth factor (HGF, Peprotech) for 5–10 days. Finally, the resultant cells were cultivated in HCM supplemented with 10 ng/ml HGF, 10 ng/ml oncostain M (OSM, Peprotech), and 10^−4^ mM dexamethasone (Dex, Sigma-Aldrich, St. Louis, MO) for another 10 days.

### Immunocytochemistry

Immunocytochemistry was performed to determine the identity of GPR125-positive cells and GPR125-negative cells by MACS, and the cells derived from human SSCs induced by the conditioned medium pursuant to the procedure described previously [44]. Briefly, cells were fixed in 4% paraformaldehyde and permeabilized in 0.4% triton-X 100 (Sigma-Aldrich) for 15 min at room temperature. After extensive washes with PBS, cells were blocked in 1% bovine serum albumin (BSA, Sigma-Aldrich) for 1 hour and incubated with primary antibodies at a dilution with 1:100 overnight at 4°C. The primary antibodies used in this study were as follows: UCHL1 (AbD Serotec), PLZF (Abcam), GFRA1 (Santa Cruz), GPR125 (Abcam), VASA (Abcam), CK8 (Santa Cruz), CK18 (Santa Cruz), CK19 (R & D System), AFP (alpha-fetoprotein) (Santa Cruz), ALB (albumin) (Santa Cruz), TRA1-60 (Chemicon), TRA1-81 (Chemicon), SSEA3 (Chemicon), AAT (alpha-1 antiproteinase) (Bethyl), CYP1A2 (Santa Cruz), and β-CATENIN (Santa Cruz). After three washes in PBS, the cells were incubated with FITC-conjugated or rhodamine-conjugated IgG secondary antibodies at a 1:200 dilution for 45 min at 34°C. DAPI (4′ -6-diamidino-2-phenylindole) was used to stain cell nuclei, and the cells were observed for epifluorescence using fluorescence microscope (Nikon Eclipse Ti-S, Nikon Corporation, Tokyo, Japan).

Immunocytochemical staining was also conducted to detect MAGEA4 expression in the freshly isolated cells pursuant to the DAB kit (Gen Tech). After permeabilization with 0.5% Triton X-100, the cells were treated with 3% hydrogen peroxide to quench endogenous peroxidase activity. Cells were blocked in 2% BSA for 30 min and incubated with an antibody to MAGEA4 at a dilution with 1:50 overnight at 4°C. After three times of washes in PBS, the cells were incubated with horse radish peroxidase-conjugated secondary antibody for 1 hour at room temperature and followed by DAB (3, 3-diaminobenzidine) as a substrate. After immunostaining, cells were counterstained with hematoxylin and observed under a light microscope (Nikon Eclipse Ti-S, Nikon Corporation, Tokyo, Japan).

### RNA extraction and reverse transcription-polymerase chain reaction (RT-PCR)

Total RNA was extracted from primary human SSCs, the transdifferentiated cells, and the fully differentiated cells using Trizol reagent (Invitrogen, Carlsbad, CA, USA). Reverse transcription (RT) of total RNA was performed using First Strand cDNA Synthesis Kit (Thermo Scientific), and PCR was performed according to the protocol described previously [45]. The primer pairs of selected genes, including *GPR125*, *UCHL1*, *GFRA1*, *PLZF*, *MAGEA4*, *RET*, *VASA*, *cytokeratin 8* (*CK8*), *CK18*, *CK7*, *CK19*, *hepatocyte nuclear factor* (*HNF4A*), *albumin* (*ALB*), *forkhead box A1 (FOXA1)*, *GATA binding protein 4* (*GATA4*), *alpha-fetoprotein* (*AFP*), b-*CATENIN*, *tyrosine aminotransferase* (*TAT*), *transferring* (*TF*), *alpha-1 antiproteinase* (*AAT*), *tryptophan 2,3-dioxygenase* (*TDO*), *cytochrome P450*, *family 1, subfamily A, polypeptide 2* (*CYP1A2*), *CYP3A4*, *CYP7A1*, *CYCLIN A2*, *CYCLIN B1*, *CYCLIN D1*, *CYCLIN E1*, *CDK1*, *CDK2*, *P21*, *P53*, and *GAPDH* were designed and listed in Table 1. The PCR reaction started at 95°C for 5 min and followed by 35 cycles: denaturation at 94°C for 30 sec, annealing at specific temperatures (Tm) as indicated in Table 1 for 45 sec, elongation at 72°C for 45 sec, and finally extension at 72°C for additional 10 min. The amplified products were separated by electrophoresis on 1.5% agarose gels, and images were captured by chemiluminescence (Chemi-Doc XRS, Bio-Rad, Hercules, CA).

**Table 1:** The primer sequences of genes used for RT-PCR

Gene	Primer sequence	Forward(5′-3′)	Product size (bp)	Tm (°C)
*UCHL1* *GPR125*	Forward Reverse Forward Reverse	CCAATGTCGGGTAGATGA CCAATGTCGGGTAGATGA TACCCTTTGGACTTGGTT TACCCTTTGGACTTGGTT	244 246	55 49
*GFRA1*	Forward Reverse	CCAAAGGGAACAACTGCCTG CGGTTGCAGACATCGTTGGA	410	58
*PLZF*	Forward Reverse	CGGTTCCTGGATAGTTTGC GGGTGGTCGCCTGTATGT	317	54
*MAGEA4*	Forward Reverse	CCGAGTCCCTGAAGATG CAGGACGATTATCAGAAGG	155	50
*RET*	Forward Reverse	CCAATGTCGGGTAGATGA CCAATGTCGGGTAGATGA	126	52
*VASA*	Forward Reverse	CCATGGGAGATGAAGATTGG CATACATTCGTCTGGGTCTAAG	425	58
*CK7*	Forward Reverse	GTGGGAGCCGTGAATATCTC GGGTGGGAATCTTCTTGTGA	250	59
*CK19*	Forward Reverse	TGACATGCGAAGCCAATATG AGTAACCTCGGACCTGCTCA	142	56
*CK8*	Forward Reverse	GACATCGAGATCGCCACCTA GCTCAGACCACCTGCATAGC	120	60
*CK18*	Forward Reverse	GAACCACGAAGAGGAAGTAAA CATCTGTAGGGCGTAGCG	361	58
*ALB*	Forward Reverse	GGTGTTGATTGCCTTTGCTC CCCTTCATCCCGAAGTTCAT	502	57
*AFP*	Forward Reverse	GCCTGTTGGAGAAATGCTG CACACCGAATGAAAGACTCG	148	56
*FOXA1*	Forward Reverse	TTCCAGACCCGTCCTAAA ACCCTCCACAAACTAGAATGTC	373	55
*CYP7A1*	Forward Reverse	GCTTGAGGCACGAGAACCT AGAAAGTCGCTGGAATGGTG	165	57
*HNF4A*	Forward Reverse	TCTGAGTGGTTGGAGGCAGT CGTAGGGATGTGGTGATTCC	125	54
*CYP3A4*	Forward Reverse	GCTGTCTCCAACCTTCACCA GCTTGCCTGTCTCTGCTTCC	110	56
*AAT*	Forward Reverse	TGAGTT CGCCTTCAGCCTAT CATCGTGAGTGTCAGCCTTG	131	52
*TDO*	Forward Reverse	GTCAAACCTCCGTGCTTCTC CTTCTTCGCTGCCTTCTACG	114	56
*TAT*	Forward Reverse	TGGAAACCTGCCTACAGACC GAAGCAATCTCCTCCCGACT	120	60
*TF* CYP1A2 *GATA4*	Forward Reverse Forward Reverse Forward Reverse	GCTTACCTGGCTCCCAATAAC TTCTTCACCACAGCAACAGC CCTCATCCTCCTGCTACCTG CTGACACCACCACCTGATTG GGAAGCCCAAGAACCTGAAT TGCCCGTAGTGAGATGACAG	101 123 169	60 60 57
*β-CATENIN*	Forward Reverse	TGGTGACAGGGAAGACATCA CCATAGTGAAGGCGAACTGC	108	56
*CYCLIN A2*	Forward Reverse	ATGTCACCGTTCCTCCTTG GGGCATCTTCACGCTCTATT	126	50
*CYCLIN B1*	Forward Reverse	GCCAATAAGGAGGGAGCA GGACCTACACCCAGCAGAAA	103	52
*CYCLIN D1*	Forward Reverse	CCCTCGGTGTCCTACTTCA CTCCTCGCACTTCTGTTCCT	108	54
*CYCLIN E1*	Forward Reverse	CTGGATGTTGACTGCCTTGA ATGTCGCACCACTGATACCC	113	50
*CDK1*	Forward Reverse	GTGCCTGAGATAACATAGAACTGG ACAACCACCACTCTGCCCTA	120	50
*CDK2*	Forward Reverse	CAGGATGTGACCAAGCCAGT TGAGTCCAAATAGCCCAAGG	125	50
*GAPDH*	Forward Reverse	AATCCCATCACCATCTTCC CATCACGCCACAGTTTCC	382	58

### Flow cytometry

Flow cytometry analysis was carried out on hepatic stem cells derived from human SSCs as described previously [46]. The cells were incubated with primary antibodies against CK18 (Santa Cruz) and CK19 (R & D System) and followed by FITC-conjugated and rhodamine-conjugated secondary antibodies. After extensive washes, the cells were analyzed by Accuri C6 flow cytometer (Accuri Cytometers, Ann Arbor, MI) and Cflow software (Accuri Cytometers). Human SSCs-derived cells without primary antibodies but with FITC-coupled and rhodamine-conjugated secondary antibodies served as a negative control.

### Albumin synthesis of hepatocytes derived from human SSCs by enzyme linked immunosorbent assay (ELISA)

Albumin synthesis of the cells derived from human SSCs was evaluated by ELISA. In brief, culture medium from human primary SSCs, mature hepatocytes derived from human SSCs, and human hepatocyte line L02 cells was collected every 2 days and stored at −80°C. The medium supernatant was concentrated with 30 kDa ultra filter units (Millipore), and ELISA assay was performed according to the manufacturer's instructions (MyBiosource). The albumin secretion was normalized to per10^5^ cells.

### Urea assay of hepatocytes derived from human SSCs

The urea nitrogen production was evaluated as described previously [26]. The cells were cultured in medium containing 2 mM ammonium chloride (Sigma-Aldrich) for 24 hours. Thereafter, the medium from human primary SSCs, mature hepatocytes derived from human SSCs, and human hepatocyte line L02 cells was collected, and urea nitrogen concentration was measured using Urea Assay Kit (Biovision, Mountain View, CA) according to the manufacturer's instructions. The urea production was normalized to per 10^5^ cells. Fresh culture medium supplemented with 2 mM ammonium chloride was used as a negative control.

### Uptake and release of indocyanine green (ICG) of hepatocytes derived from human SSCs

The ICG uptake and release was conducted according to the method as described previously [26]. Hepatocytes derived from human SSCs were incubated with 1 mg/ml ICG (Sigma-Aldrich) for 30 min at 37°C. After extensive washes with PBS, the cellular uptake of ICG was photographed under the microscope. The cells were cultured in freshly HCM for another 20 hours at 37°C, and the release of cellular ICG staining was examined. Human hepatocyte line L02 cells and human germline stem cell line [22] were used as a positive control and a negative control, respectively.

### Statistical analysis

All experiments were performed independently at least 3 times. All the values were presented as mean ± SEM. Statistical analyses were conducted using the analysis of variance (ANOVA) and a 2-tailed *t*-test, and *P* values of less than 0.05 (*p* < 0.05) were considered statistically significant differences.
